# FAM135B sustains the reservoir of Tip60‐ATM assembly to promote DNA damage response

**DOI:** 10.1002/ctm2.945

**Published:** 2022-08-17

**Authors:** Kai Zhang, Qingnan Wu, Wenzhong Liu, Yan Wang, Lianmei Zhao, Jie Chen, Haoyu Liu, Siqi Liu, Jinting Li, Weimin Zhang, Qimin Zhan

**Affiliations:** ^1^ Key Laboratory of Carcinogenesis and Translational Research (Ministry of Education/Beijing) Laboratory of Molecular Oncology Peking University Cancer Hospital & Institute Beijing China; ^2^ Institute of Cancer Research Shenzhen Bay Laboratory Shenzhen China; ^3^ Research Unit of Molecular Cancer Research Chinese Academy of Medical Sciences Beijing China; ^4^ Department of Oncology Cancer Institute Peking University Shenzhen Hospital Shenzhen Peking University‐Hong Kong University of Science and Technology (PKU‐HKUST) Medical Center Shenzhen China; ^5^ Peking University International Cancer Institute Beijing China; ^6^ Research Center The Fourth Hospital of Hebei Medical University Shijiazhuang Hebei China

## Abstract

**Background:**

Recently, the mechanism by which cells adapt to intrinsic and extrinsic stresses has received considerable attention. Tat‐interactive protein 60‐kDa/ataxia–telangiectasia‐mutated (TIP60/ATM) axis‐mediated DNA damage response (DDR) is vital for maintaining genomic integrity.

**Methods:**

Protein levels were detected by western blot, protein colocalisation was examined by immunofluorescence (IF) and protein interactions were measured by co‐immunoprecipitation, proximity ligation assay and GST pull‐down assays. Flow cytometry, comet assay and IF assays were used to explore the biological functions of sequence similarity 135 family member B (FAM135B) in DDR. Xenograft tumour, FAM135B transgenic mouse models and immunohistochemistry were utilised to confirm in vitro observations.

**Results:**

We identified a novel DDR regulator FAM135B which could protect cancer cells from genotoxic stress in vitro and in vivo. The overexpression of FAM135B promoted the removal of γH2AX and 53BP1 foci, whereas the elimination of FAM135B attenuated these effects. Consistently, our findings revealed that FAM135B could promote homologous recombination and non‐homologous end‐joining repairs. Further study demonstrated that FAM135B physically bound to the chromodomain of TIP60 and improved its histone acetyltransferase activity. Moreover, FAM135B enhanced the interactions between TIP60 and ATM under resting conditions. Intriguingly, the protein levels of FAM135B dramatically decreased following DNA damage stress but gradually increased during the DNA repair period. Thus, we proposed a potential DDR mechanism where FAM135B sustains a reservoir of pre‐existing TIP60‐ATM assemblies under resting conditions. Once cancer cells suffer DNA damage, FAM135B is released from TIP60, and the functioning pre‐assembled TIP60‐ATM complex participates in DDR.

**Conclusions:**

: We characterised FAM135B as a novel DDR regulator and further elucidated the role of the TIP60‐ATM axis in response to DNA damage, which suggests that targeting FAM135B in combination with radiation therapy or chemotherapy could be a potentially effective approach for cancer treatment.

## Graphical abstract

1. High levels of FAM135B are more resistant to genotoxic stress in vitro and in vivo.
2. FAM135B enhanced the interactions between TIP60 and ATM in resting conditions.
3. FAM135B will be released from the pre‐assemble TIP60‐ATM module for a rapid DNA damage response.

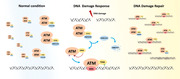

## 1 INTRODUCTION

The maintenance of genomic stability is continuously disturbed by intrinsic stresses from DNA replication and extrinsic environmental factors, such as chemotherapy agents and ionising radiation.^1^, ^2^, ^3^ These stresses induce DNA lesions that later evolve into genomic instability. To avoid stress‐induced DNA damage, cells activate a range of DNA repair signalling pathways to restore their genome to its original state, including nucleotide excision repair, base excision repair and DNA double‐strand break (DSB) repair: homologous recombination (HR), non‐homologous end‐joining (NHEJ).^4^, ^5^, ^6^, ^7^ Nonetheless, the DNA damage repair machineries are far from fully understood. Therefore, exploring novel DNA damage response (DDR) factors and repair mediators is urgently needed.

The histone acetyltransferase (HAT) Tat‐interactive protein 60‐kDa (TIP60) participates in the cellular activities of several tumours, including cell apoptosis, tumour invasion and, pertinent to this study, DNA damage repair.^8^, ^9^, ^10^, ^11^ Following DNA damage, TIP60 is recruited to the damaged sites and bound to the methylated histone. Subsequently, ATM is acetylated at the lysine 3016 site, which rapidly activates itself and promotes a series of phosphorylation events.^12^, ^13^, ^14^, ^15^ The phosphorylation pathway is then translated into the DNA repair pathway via ATM‐dependent phosphorylation of H2AX (γH2AX) that mediates further recruitment of other DNA repair machinery.^16^, ^17^, ^18^, ^19^ Furthermore, previous studies have revealed that TIP60 plays an essential role in DNA damage‐induced cell cycle arrest, P53‐mediated cell cycle regulation and Aurora B‐mediated accurate mitosis and chromosomal stability.^20^, ^21^, ^22^, ^23^

Recently, many proteins have been implicated in the DNA damage repair functions of TIP60. A previous study showed that straight interactions between TIP60 and histone trimethylated H3 on lysine 9 (H3K9me3) could improve the acetyltransferase activity of TIP60.^24^ It has been proposed that ATF2 regulates the expression levels of TIP60 subsequently affecting ATM activity.^25^ Additionally, E3 ubiquitin–protein ligase RNF8 has been demonstrated to facilitate the efficient repair of DSB through TIP60.^26^ It seems that the functions of TIP60 in DNA repair are affected by several factors, and discovering novel mediators of TIP60 acetyltransferase activity will extend our current knowledge of DNA repair.

In a previous study, our group first identified that FAM135B is a potential driver gene of oesophageal squamous cell carcinoma (ESCC) and is frequently mutated and amplified in ESCC patients.^27^, ^28^ As FAM135B is a gene with unknown functions, our team then conducted in‐depth research on its function and underlying molecular mechanisms and reported, for the first time, that FAM135B promotes tumour initiation and progression through the GRN/AKT/mTOR pathway.^29^ Interestingly, our preliminary study demonstrated that FAM135B was located both in the nucleus and cytoplasm, and its overexpression protected tumour cells from agent and γ‐irradiation (IR)‐induced DNA damage, confirming its roles in DDR. Similarly, a recent study demonstrated that the knock‐down of FAM135B sensitised ESCC cells to IR.^30^ Additionally, FAM135B is predicted to interact with TIP60 in online protein–protein interaction (PPI) tools, further linking FAM135B to genome fidelity. These observations have raised several interesting questions, such as what the molecular mechanism behind these phenomena may be, and whether FAM135B regulates TIP60 thereby taking part in DDR.

In this study, we explored the biological functions and underlying mechanism of FAM135B in DDR. Our study demonstrated that tumour cells with a high level of FAM135B acquired resistance to chemotherapy and IR‐induced DNA damage. Furthermore, FAM135B was identified as a new TIP60 regulator that promoted the HAT activity of TIP60 by physically interacting with TIP60. The overexpression of FAM135B led to enhanced interactions between TIP60 and ATM, thus promoting ATM activation following DNA damage. These results will expand our understanding of DNA damage repair and may provide new therapeutic targets in clinical practises. Collectively, these findings provide a new avenue for understanding DNA repair mediated by the FAM135B‐TIP60‐ATM pathway and highlight the novel function of FAM135B in chemotherapy and radiotherapy resistance.

## 2 MATERIALS AND METHODS

### 2.1 and drugs

The following specific antibodies were used in this study: Anti‐γH2AX (#2577), anti‐γ‐H2AX (#9718) and anti‐GAPDH (#51332), anti‐H4K8ac (#2594), anti‐ATM (#2873), anti‐ATM (#92356), anti‐pATM (#5883S), anti‐ATR (#13934), anti‐pATR (#2853), anti‐CHK1(#2360), anti‐pCHK1 (#2348), anti‐CHK2 (#6334), anti‐pCHK2 (#2197), anti‐FLAG (8146), anti‐MYC (71D10) and anti‐TIP60 (#12058) were all purchased from Cell Signaling Technology (Boston, MA). Anti‐TIP60 (#10398) was purchased from Abnova. Anti‐ATM (#ET‐1606‐20) was purchased from Human Technology. Anti‐FAM135B (SAB2104963) was purchased from Sigma‐Aldrich (Merck Darmstadt, Germany). Anti‐FLAG (ab205606) and anti‐53BP1 (ab36823) antibodies were acquired from Abcam (England); anti‐GST (HT601) was acquired from Santa Cruz (Dallas, USA); bleomycin (BLM, radiomimetic drug), etoposide (ETO) and cisplatin (CDDP, a chemotherapy drug that can cause DNA double‐chain rupture as a DNA damage inducer) purchased from Selleck (America). We describe all the antibodies and drugs in Tables S1 and S2.

### 2.2 Cell culture and treatment

U2OS and HEK‐293T cells were maintained in Dulbecco's Modified Eagle Medium supplemented with 10% fetal bovine serum (FBS, Gibco, USA) in a humidified incubator with 5% CO2. YES2, KYSE30, KYSE450 and KYSE510, KYSE30‐EV and KYSE30‐FAM13B cells were all cultured in RPMI‐1640 (Gibco, USA) with 10% FBS.

### 2.3 Plasmids and siRNA transfection

FLAG‐FAM135B plasmid and control plasmid were purchased from Tianyi Huiyuan Life Science & Technology Inc. MYC‐TIP60 plasmid, four MYC‐TIP60 truncated fragments, GST‐FAM135B plasmid and GST plasmid were purchased from Mailgene Biosciences Co., Ltd. In addition, the pDRGFP plasmid was kindly provided by Dr. Maria Jasin (Addgene).^31^ PimEJ5GFP plasmid was kindly provided by Dr. Jeremy Stark (Addgene).^32^ ISceI‐GR‐RFP plasmid was kindly provided by Dr. Tom Misteli (Addgene).^33^ Plasmids were transfected into HEK‐293T and other cancer cells using Lipofectamine 2000 (Invitrogen). Cancer cells were transiently transfected with specific siRNA or negative control siRNA using the Lipofectamine 2000 reagent. We describe all the plasmids, primers and siRNA in a Tables S3–S5.

### 2.4 Cell proliferation assay

For the 3‐(4,5‐dimethylthiazol‐2‐yl)‐5‐(3‐carboxymethoxyphenyl)‐2‐(4‐sulfophenyl)‐2H‐tetrazolium (MTS) assay, indicated cells were all plated in 96‐well plates (3000–5000 cells/well), and the cells were treated with the indicated concentrations of the indicated drug. The growth capacity of these cells was measured by MTS assay (Promega). As for the colony formation assay, 500–2000 tumour cells were plated into 6‐well plates and incubated for 1 day with the indicated concentrations of different drugs at 37°C, then refreshed with a fresh medium. After culturing for 7–10 days, these cells were fixed and stained with crystal violet. The numbers of colonies were counted. The assay was carried out in every cell line in triplicate. Data were presented as the mean ± SD of three independent experiments.

### 2.5 Homologous recombination and non‐homologous end‐joining repair assay

KYSE30 cells were transfected with either a FAM135B or empty control. Then the transfected cells were co‐transfected with pDRGFP or pimEJ5GFP plasmid with ISceI‐GR‐RFP using Lipofectamine 2000. After 72 h, flow cytometric analysis was performed on Acurri C6. Then we get the percentage of GFP‐positive cells through FACS. U2OS cells were transfected with either an FAM135B or a vector control. Then the transfected cells were co‐transfected with pDRGFP or pimEJ5GFP plasmid with ISceI‐GR‐RFP using Lipofectamine 2000. After 72 h, flow cytometric analysis was performed on Acurri C6. Then we get the percentage of GFP‐positive cells through FACS. The experiments were performed in triplicate.^34^, ^35^, ^36^

### 2.6 GST pull‐down assay

The recombinant indicated plasmid transfer to BL21 (DE3) strains and was cultured overnight at 37°C overnight. We used IPTG to induce GST‐FAM135B fusion protein. We collected the GST‐FAM135B fusion protein by glutathione *S*‐transferase bead in accordance with the manufacturer's guidelines (Bersin bio). HEK‐293T cells with MYC‐TIP60 full length (FL) or truncated fragment plasmids transfected were collected using the GST pull‐down KIT (Bersin bio) to perform GST pull‐down assay in accordance with the manufacturer's guidelines.

### 2.7 Histone acetyltransferase (HAT) activity assay

The HAT Activity Assay was carried out by utilising HAT assay kits (ab65352, Abcam) in accordance with the manufacturer's instructions. The nuclear protein MYC‐TIP60 complex from these indicated cells was extracted using anti‐MYC magnetic beads, and the OD value for spectrophotometric measurements was read at 440 nm.^37^, ^38^

### 2.8 Immunofluorescence (IF)

As for immunofluorescence (IF), indicated cells were grown on confocal plates, then we fixed them with methyl alcohol or 4% formaldehyde for about 20 min. Cells were blocked in 10% serum for 30 min. These cells were incubated with the suitable antibody at 4°C overnight. These cells were washed by PBS and treated with a secondary antibody Alexa Fluor 488 or Alexa Fluor 594 (ZSGB‐BIO: 0516,0512), then incubated in DAPI for 5 min. Pictures were caught and envisaged by a confocal microscope (63 × oil immersion; Leica ST2, Leica, Germany).

### 2.9 Comet assay

The comet assay was carried out by utilising the Comet Assay Kit (Trevigen, Gaithersburg, MD) in accordance with the manufacturer's instructions. Pictures were caught and envisaged by a confocal microscope. Comet Score software (TriTek, Sumerduck, USA) measured the tail moment.

### 2.10 Western blotting and immunoprecipitation (IP)

The indicated cells were harvested in RIPA lysis buffer. Subsequently, the proteins proposed by RIPA lysis buffer. Then the sample proteins were incubated with 2 μg indicated antibodies or IgG antibodies with protein A/G Sepharose beads and NT‐2 buffer (50‐mM Tris–HCl: pH 7.4, 150‐mM NaCl, 1‐mM MgCl_2_, .25% NP40) together at 4°C overnight.

Then we washed the beads with NT‐2 buffer, and the samples were detected with antibodies as indicated by western blotting. As for western blotting, the chemiluminescence marks were caught using Amersham Imager 600 (GE, America).

### 2.11 Xenograft studies

We bought female BALBC/nude mice from Vital River Laboratories (Beijing, China). About 1× 10^7^ KYSE30‐EV or KYSE30‐FAM135B cells were all injected into the left leg of these nude mice (*n* = 8, respectively). When the tumour volumes arrived at about 100 mm^3^, 5‐mg/kg CDDP was intraperitoneally injected twice a week for 24 days. Tumour length and width were measured twice a week, and the tumour volumes were calculated: (length) × (width)^2^ /2. Lastly, the mice were euthanasia. All experiments were carried out in accordance with national and institutional animal health and well‐being policies. All the animal assays were approved by the Peking University Cancer Hospital Animal Care and Use Committee (Approval no. EAEC 2020‐08).

### 2.12 FAM135B transgenic mice assay

FAM135B transgenic mice (FAM135Btg) were generated by our group previously.^29^ We used mouse tail to detect the genotype with primers: 5′‐TCCCCAAGACCGTTATGTGC‐3′ (forward) and 5′‐GCCGCTACTTGTCATCGTCA‐3′ (reverse). 6‐8‐week‐old FAM135Btg and wide‐type mice were used in this assay. All mice were housed in ventilated cages for 12‐h day and night, with free access to drink water. Wild‐type (*n* = 6) and FAM135Btg (*n* = 6) female mice were divided into two groups, one as a negative control, five underwent irradiations and, subsequently, mice were euthanised at the following time points: 0, 1, 6, 12 and 24 h, and the relevant tissues were taken for experiments.

### 2.13 Immunohistochemistry and staining score

The tissues were first embedded in paraffin; then paraffin sections are dehydrated, antigen repair in citric acid repair solution, and 10% goat serum was incubated for 30 min at room temperature, then add anti‐γH2AX primary antibody on the sections and incubate the sections flat in the humidified box at 4°C for overnight. The following day, add a second antibody at room temperature for 50 min. Add tyramine–biotin to the tissue and incubate at room temperature for 20 min. Lastly, DAB staining and reflected nuclei. The immunohistochemistry (IHC) staining scores were according to the IRS scoring system of 0–12 points. Two clinicians used a double‐blind approach to measure the grade of the intensity of staining and the estimated percentage of positive cells. The scores of γH2AX, and FAM135B IHC staining, were defined as the intensity of staining (0, negative; 1, weak; 2, medium; 3, strong) multiplied by the measured positive cells rank. The estimated percentage of positive cells was ranked as 0 (negative‐10%), 1 (10%–25%), 2 (26%–50%), 3 (51%–75%) and 4 (>75%) in each tissue. Five views were taken per tissue.^39^, ^40^

For IHC of ESCC tissues, tissues were subjected to immunostaining using antibodies FAM135B. These retrospective ESCC specimens from patients after platinum‐based neoadjuvant therapy were obtained from the Fourth Hospital of Hebei Medical University, Shijiazhuang, Hebei, China. Written informed consent was obtained from all patients prior to the study. The use of the clinical specimens for research purposes was approved by the Institutional Research Ethics Committee of The Fourth Hospital of Hebei Medical University (2017MEC112). The detailed clinicopathological characteristics of all specimens are summarised in Table S6.

### 2.14 Proximity ligation assay (PLA)

The protein–protein interactions in situ within 30 nm of each protein were detected by a proximity ligation assay (PLA) kit (DUO92101, Sigma‐Aldrich). The Duolink PLA kit was used in accordance with the manufacturer's instructions. For the quantification of PLA fluorescence signals, pictures were caught and envisaged by a confocal microscope (63 × oil immersion; Leica ST2, Leica, Germany). Red dots of PLA signals were measured in the cell.

### 2.15 Statistical analysis

Student's *t*‐test and one‐way ANOVA analyses of variance with pairwise comparisons were used to determine the differences between control and treatment groups. All experiments were individually carried out three times, and the mean values and standard deviations were calculated. Statistical analysis was performed using GraphPad Prism 7 (GraphPad, San Diego, CA). The results were presented as the mean ± SD of three independent tests. The statistical significance of these results: *p < .05, **p < .01 and ***p < .001.

## 3 RESULTS

### 3.1 FAM135B protects tumour cells from genotoxic stress in vitro and in vivo

**FIGURE 1:**
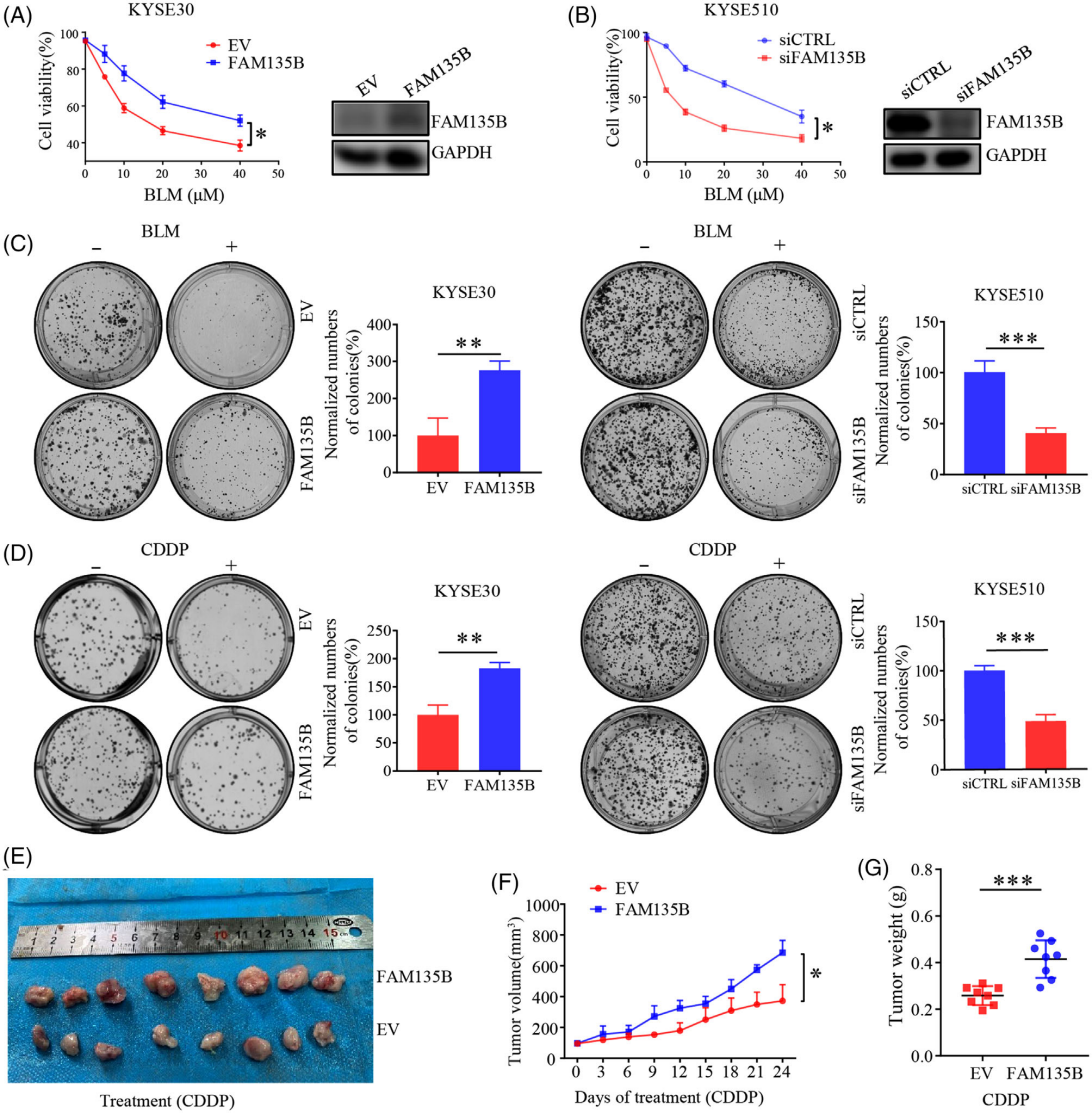
**FAM135B mediates tumour cells resistance to DNA damage in vitro and in vivo**. (A and B) Cell growth curve examined by 3‐(4,5‐dimethylthiazol‐2‐yl)‐5‐(3‐carboxymethoxyphenyl)‐2‐(4‐sulfophenyl)‐2H‐tetrazolium (MTS) assay. KYSE30 cells (A) were transfected with FLAG‐FAM135B or an empty vector and KYSE510 cells (B) were transfected with FAM135B (siFAM135B) or control siRNA (siCTRL). After 48 h, a part of the cells was seeded in 96‐well plates and treated with different concentrations of bleomycin (BLM). Another part of the cells was used for western blotting to detect protein expression efficiency. The MTS assay was used to monitor cell proliferation rate. (C and D) Colony formation assays were performed on tumour cells transfected with an empty vector or FAM135B (in KYSE30 cells) and siFAM135B or siCTRL (in KYSE510 cells) and treated with different kinds of DNA damaging drugs, including Cisplatin (CDDP, 100 nM; C) or BLM (1 μM; D) for 24 h, then refreshed with fresh medium. After 7–10 days, cells were fixed and stained. Data are presented as mean ± SD. Images are the representative of from the collation of three independent experiments. Quantification of the number of normalised colonies was presented in a histogram. (E–G) A xenograft model was used for evaluating the effect of FAM135B on CDDP response. Xenograft tumours from KYSE30 cells transfected with an empty vector or FAM135B were treated with 5‐mg/kg CDDP when they were approximately 100 mm^3^. (E) The images of xenograft tumours dissected at the endpoint. (F) The xenograft tumour growth curve. (G) The xenograft tumour mass weight at the endpoint

Given that FAM135B is a potential driver of ESCC, we sought to determine whether FAM135B was implicated in chemotherapy sensitivity. Initially, we observed relatively high expression levels of FAM135B in YES2, KYSE450 and KYSE510 cells, but low levels in KYSE30 (Figure S1A). Thus, we transfected KYSE30 cells with FAM135B and empty vectors and found that cells with overexpressed FAM135B were more resistant to DNA damage‐induced agent BLM (Figure 1A), whereas the knock‐down of FAM135B in KYSE510 cells sensitised cells to BLM (Figure 1B). Consistently, colony formation assays confirmed that the overexpression of FAM135B increased the colony numbers after BLM and CDDP treatments, whereas the knock‐down of FAM135B displayed decreased colony numbers (Figure 1C,D). Similar results were observed under etoposide (ETO) treatment where FAM135B mediated resistance to ETO (Figure S1B,C). To further validate these findings, we tested the drug response in a xenograft model. As expected, xenografts with FAM135B overexpression were more resistant to CDDP (Figure 1E–G). Overall, these results suggested that FAM135B plays a role in mediating resistance to DNA damage agents.

### 3.1 FAM135B regulates DNA damage response and facilitates DNA repair

**FIGURE 2:**
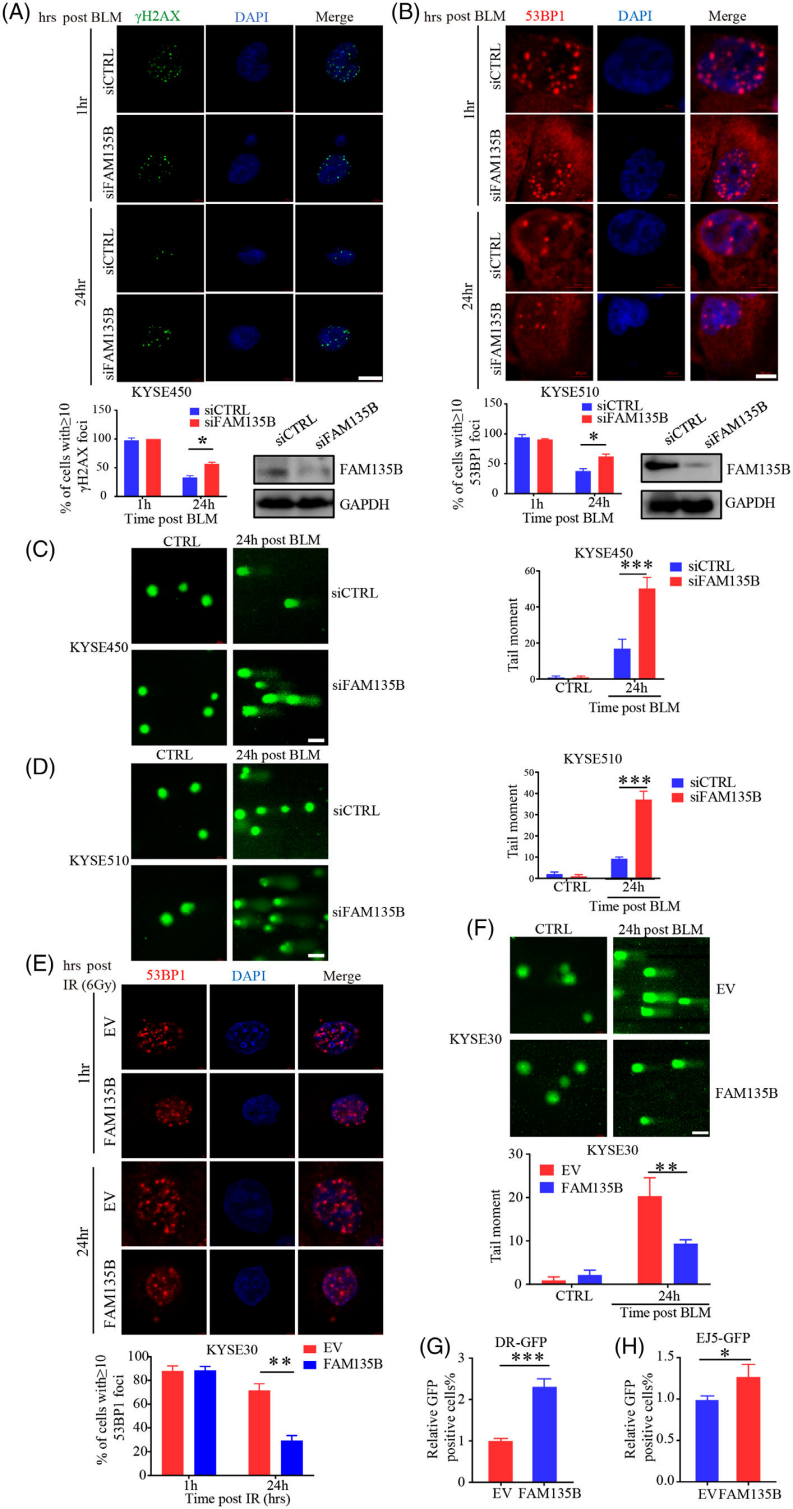
**FAM135B regulates the impact of DNA damage and accelerates DNA repair** (A and B) Immunofluorescence analysis of the formation of γH2AX and 53BP1 foci. KYSE450 cells were transient transfected with FAM135B siRNA (siFAM135B) or control siRNA (siCTRL). After 48 h, a western blot was performed to evaluate knockdown efficiency. The KYSE450‐siCTRL or KYSE450‐siFAM135B (A) and KYSE510‐siCTRL or KYSE510‐siFAM135B cells (B) were treated with 10‐μM BLM for 12 h then released into fresh media without BLM to enable DNA damage response at different time points. Cells were fixed at 1 or 24 h after the removal of BLM and were immunostained with anti‐γH2AX (green) or anti‐53BP1 antibodies (red) with DAPI (blue). Top: representative images, bottom: Quantification data of γH2AX/53BP1 foci; more than 50 cells were measured. Scale bar = 10 μm. (C and D) Representative images and quantification of comet assay. KYSE450‐siCTRL or KYSE450‐siFAM135B (C) and KYSE510‐siCTRL or KYSE510‐siFAM135B cells (D) were treated with 10‐μM BLM for 12 h, then released into fresh median without BLM to enable DNA damage response for 24 h. Left: representative images, right: quantification data. Scale bar = 40 μm. (E) Immunofluorescence analysis of the formation of 53BP1 foci. KYSE30 cells were transfected with FLAG‐FAM135B or empty vectors for 48 h then subjected to 6 Gy of γ‐irradiation then permitted to DNA damage response at different time points. Top, the representative image of 53BP1 foci, scale bar = 10 μm. Bottom, the quantification data. (F) Representative images and quantification of comet assay. KYSE30 cells were transfected with FLAG‐FAM135B or an empty vector for 48 h and then were treated with 10‐μM BLM for 12 h, then released into fresh medium without BLM for permitting DNA damage response for 24 h. Top, representative images of the comet assay, right, quantification data. Scale bar = 40 μm. (G) Homologous recombination (HR) rate was monitored by pDRGFP reporter Assay KYSE30 cells co‐transfected with FLAG‐FAM135B or empty vectors with pDRGFP and ISceI‐GR‐RFP. After 72 h, cells were examined for GFP‐positive fluorescence cells by flow cytometry. Histogram showed relative GFP‐positive fluorescence compared to the control cells. (H) Non‐homologous end‐joining (NHEJ) DNA repair rate were monitored by pimEJ5GFP reporter assays. KYSE30 cells were co‐transfected with FLAG‐FAM135B or empty vectors with pimEJ5GFP and ISceI‐GR‐RFP. After 72 h, cells were examined for GFP‐positive fluorescence cells by flow cytometry. Histogram showed the relative GFP‐positive fluorescence compared to the control cells.

Next, we sought to elucidate whether FAM135B was implicated in DDR. We first transfected KYSE450 cells with FAM135B siRNA or control siRNA and treated cells with 10‐μM BLM for 12 h to induce DNA damage. Later, we refreshed the cell culture media without BLM to permit DNA repair. Following IF staining with γH2AX, a well‐known DNA damage marker,^41^ we found no significant difference in γH2AX foci numbers between the FAM135B knock‐down and control groups at the early repair stage (Figure 2A). However, there were more γH2AX foci in the cells that knocked down FAM135B after releasing for 24 h (Figure 2A). Similarly, the 53BP1 foci, which indicate the DNA repair apparatus, were also more apparent in FAM135B silenced cells (Figure 2B), suggesting that FAM135B deficiency impairs the elimination of DNA lesions. To further confirm the role of FAM135B in DNA injury repair, we employed alkaline comet experiments. After releasing from BLM, the tail moments were still maintained at 24 h in FAM135B knock‐down cells (KYSE510 and KYSE450) compared to the control cells (Figure 2C,D). Similarly, the IF assay for 53BP1 and comet assay demonstrated that overexpressed FAM135B in U2OS cells had fewer 53BP1 foci (Figure S2A,B) and shorter DNA tail moments under the same experimental conditions mentioned earlier (Figure S2C). Consistently, KYSE30 cells with overexpressed FAM135B had fewer 53BP1 foci 24 h after IR (Figure 2E) and shorter DNA tail moments 24 h after BLM‐induced DNA damage (Figure 2F), indicating that the overexpression of FAM135B endows cells with stronger DNA damage repair abilities.

HR and NHEJ are two major pathways used to repair DSBs.^5^ Thus, we used pDRGFP or pimEJ5GFP and ISceI‐GR‐RFP endonuclease plasmids to evaluate HR/NHEJ‐mediated repair efficiency. The ISceI‐GR‐RFP endonuclease plasmid with pDRGFP plasmid indicated HR and with the pimEJ5GFP plasmid indicated NHEJ. KYSE30‐EV cells or KYSE30‐FAM135B co‐transfected pDRGFP or pimEJ5GFP with ISceI‐GR‐RFP plasmids. After 72 h, the GFP‐positive cells were analysed by flow cytometry. We found that the overexpression of FAM135B could display more GFP‐positive cells (Figure 2G,H). U2OS cells were co‐transfected with FAM135B‐FLAG (or empty vector) and the ISceI‐GR‐RFP endonuclease plasmid with pDRGFP or pimEJ5GFP. After 72 h, the GFP‐positive cells were analysed by flow cytometry. We found that the overexpression of FAM135B promoted the HR and NHEJ repair pathways (Figure S2D,E). These findings suggest that FAM135B promotes the repair of DSBs.

### 3.2 FAM135B coordinates ATM and its downstream effectors in response to DNA damage

**FIGURE 3:**
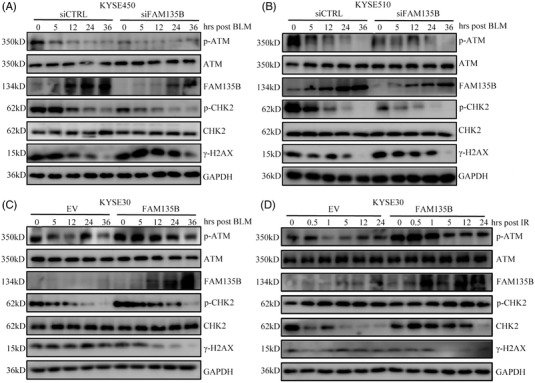
**FAM135B affects ATM activity in DNA repair**. (A and B) KYSE450 (A) and KYSE510 (B) cells were transfected with siFAM135B or siCTRL for 48 h and subsequently treated with 10‐μM BLM for 12 h, then cells were harvested at the indicated time point and subjected to western blots with indicated antibodies. (C and D) KYSE30 cells were transfected with FAM135B‐FLAG or empty vector (EV) for 48 h and subsequently treated with 10‐μM BLM for 12 h (C) or 6‐Gy IR (D), then cells were harvested at the indicated time points and subjected to western blot with indicated antibodies.

As FAM135B was implicated in DNA damage and repair, we aimed to determine how FAM135B regulated the DDR pathway. It is well documented that ATM signalling is the most important in the DDR pathway.^16^ Thus, we investigated the activities of ATM and its downstream effectors upon FAM135B elimination or overexpression in ESCC cell lines. As expected, the phosphorylation levels of ATM (Ser1981) and its downstream effector, CHK2, gradually decreased overtime post BLM treatment release or IR, indicating that cells had properly executed the DNA repair function (Figures 3A–D and S2F). The phosphorylation levels of ATM and CHK2 were notably compromised in FAM135B knock‐down cells (Figure 3A,B), whereas they persisted in FAM135B overexpression cells (Figures 3C,D and S2F), suggesting that FAM135B can sustain their phosphorylation. In contrast, γH2AX levels gradually decreased overtime after BLM treatment or IR (Figures 3A–D and S2F). We observed that the decrease in γH2AX was delayed in the FAM135B elimination group compared to the control, whereas it was removed more rapidly in the FAM135B overexpression group (Figures 3A–D and S2F), indicating that FAM135B can enhance DNA repair efficiency. Surprisingly, we found that the protein levels of FAM135B were gradually increased after BLM treatment release or IR (Figures 3A–D and S2F). Overall, these results further support the notion that FAM135B regulates ATM signalling to deal with genotoxic stress.

### 3.3 FAM135B physically interacts with TIP60

**FIGURE 4:**
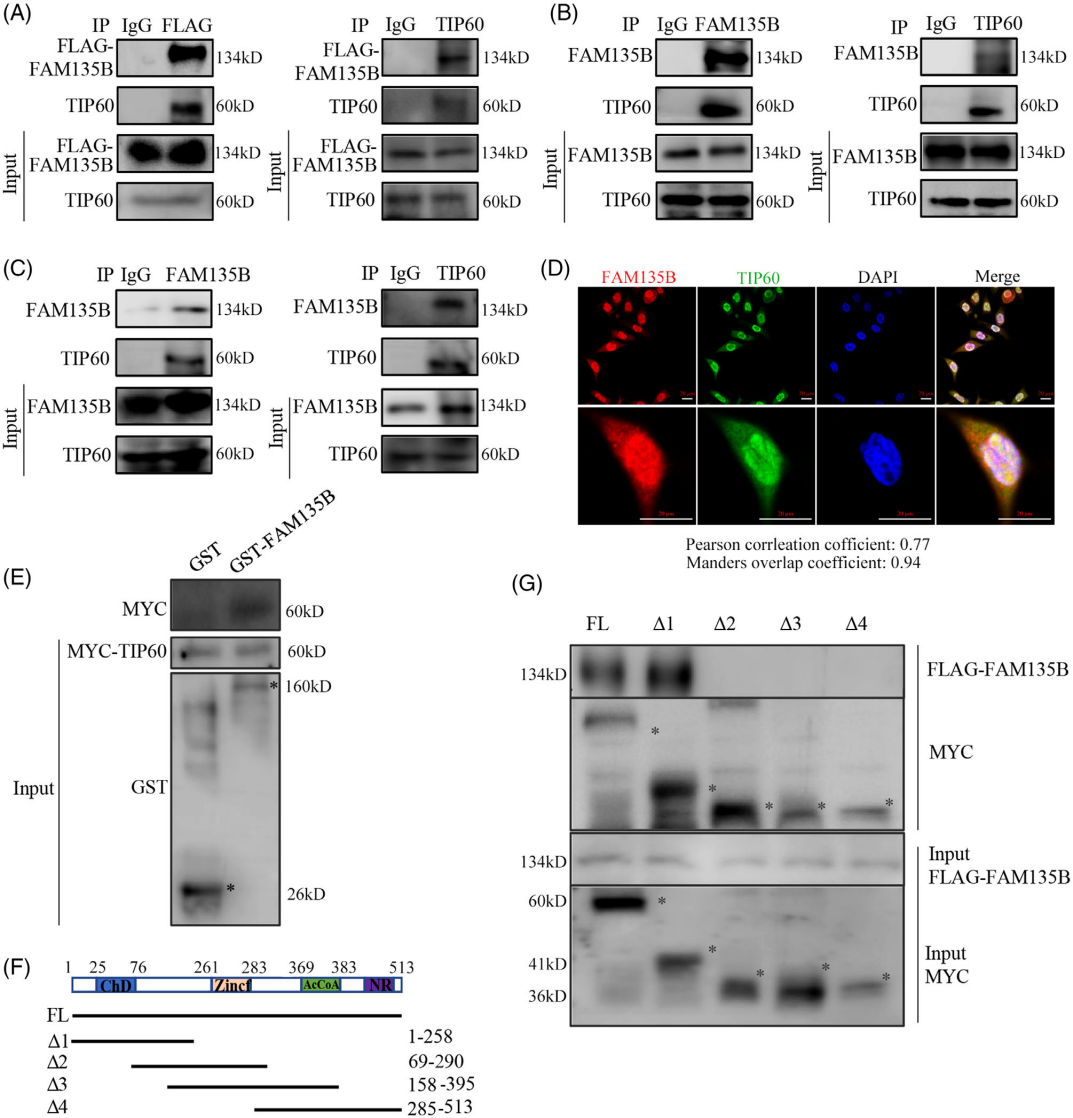
**FAM135B physically interacts with TIP60**. (A–C) Co‐immunoprecipitation (Co‐IP) assays identified the interaction between FAM135B and TIP60. Stably expressed FAM135B‐FLAG in KYSE30 cells was used for Co‐IP assays by anti‐FLAG (left panel) and anti‐TIP60 (right panel) antibodies (A) or anti‐FAM135B (left panel) and anti‐TIP60 (right panel) antibodies (B). Endogenous interactions between the TIP60 and FAM135B were confirmed by Co‐IP assays in KYSE510 cells using anti‐FAM135B and anti‐TIP60 antibodies (C). IgG was used as the negative control. (D) The immunofluorescence (IF) assay revealed the colocalisation of FAM135B and TIP60 in the nuclei of KYSE30‐FAM135B cells. Scale bar = 20 μm. (E) The GST pull‐down assay confirmed the interaction between FAM135B and TIP60. GST‐FAM135B fusion proteins were purified and incubated with HEK‐293T cell lysates that expressed MYC‐TIP60 and analysed by SDS–PAGE western blotting (* indicates correct band). Purified GST proteins served as negative controls. (F and G) FAM135B bound to the chromodomain of TIP60. Schematic depiction of serial truncated deletion of MYC‐TIP60 was constructed (F). HEK293T cells were co‐transfected with FAM135B‐FLAG and different MYC‐TIP60 fragments (full length [FL] and Δ1–4) for 48 h, and then cells were harvested and supplied for Co‐IP assay. * indicates correct band, aa, amino acids

Our previous data confirmed that FAM135B is implicated in DDR. Additionally, FAM135B was predicted to interact with TIP60, the critical modulator of ATM signalling, in the IID and HPRD public database. Thus, it is reasonable to speculate that the function of FAM135B on DDR depends on TIP60‐ATM. To verify this hypothesis, we performed a co‐immunoprecipitation (Co‐IP) assay to determine the interactions between FAM135B and TIP60. Unsurprisingly, anti‐FAM135B and anti‐TIP60 antibody both immunoprecipitated each other (Figure 4A,B), and these observations were confirmed by the exogenous expression of FAM135B‐FLAG and subsequent Co‐IP by anti‐FLAG or anti‐TIP60 (Figure 4C). Additionally, the IF assay demonstrated the colocalisation of FAM135B and TIP60 mainly in the cell nucleus (Figure 4D). To determine whether FAM135B physically interacts with TIP60 in vitro, we incubated purified recombinant GST‐FAM135B and GST proteins (as negative controls) with MYC‐TIP60 expressed HEK293T cell lysates. The pull‐down assay showed that FAM135B is bound to TIP60 (Figure 4E). Then we sought to define the domains required for the FAM135B‐TIP60 interaction. To this end, we constructed and purified FL MYC‐TIP60 (FL, 1‐513 aa) and several fragments, including Δ1 (1‐258 aa, containing the chromodomain, ChD), Δ2(69‐290 aa, harbouring the Zinc finger domain, ZinF), Δ3 (158‐395 aa, including the ZinF and acetyl‐CoA‐binding domain, AcCoA) and Δ4 (285‐513 aa, including the AcCoA and NR domain, Figure 4F). The results revealed that GST‐FAM135B could only pull down the full‐length TIP60 and the Δ1 fragment, suggesting that FAM135B bound to the ChD of TIP60. Collectively, these data suggest that FAM135B physically interacts with TIP60.

### 3.5 FAM135B enhances the TIP60 HAT activity and promotes ATM activation

**FIGURE 5:**
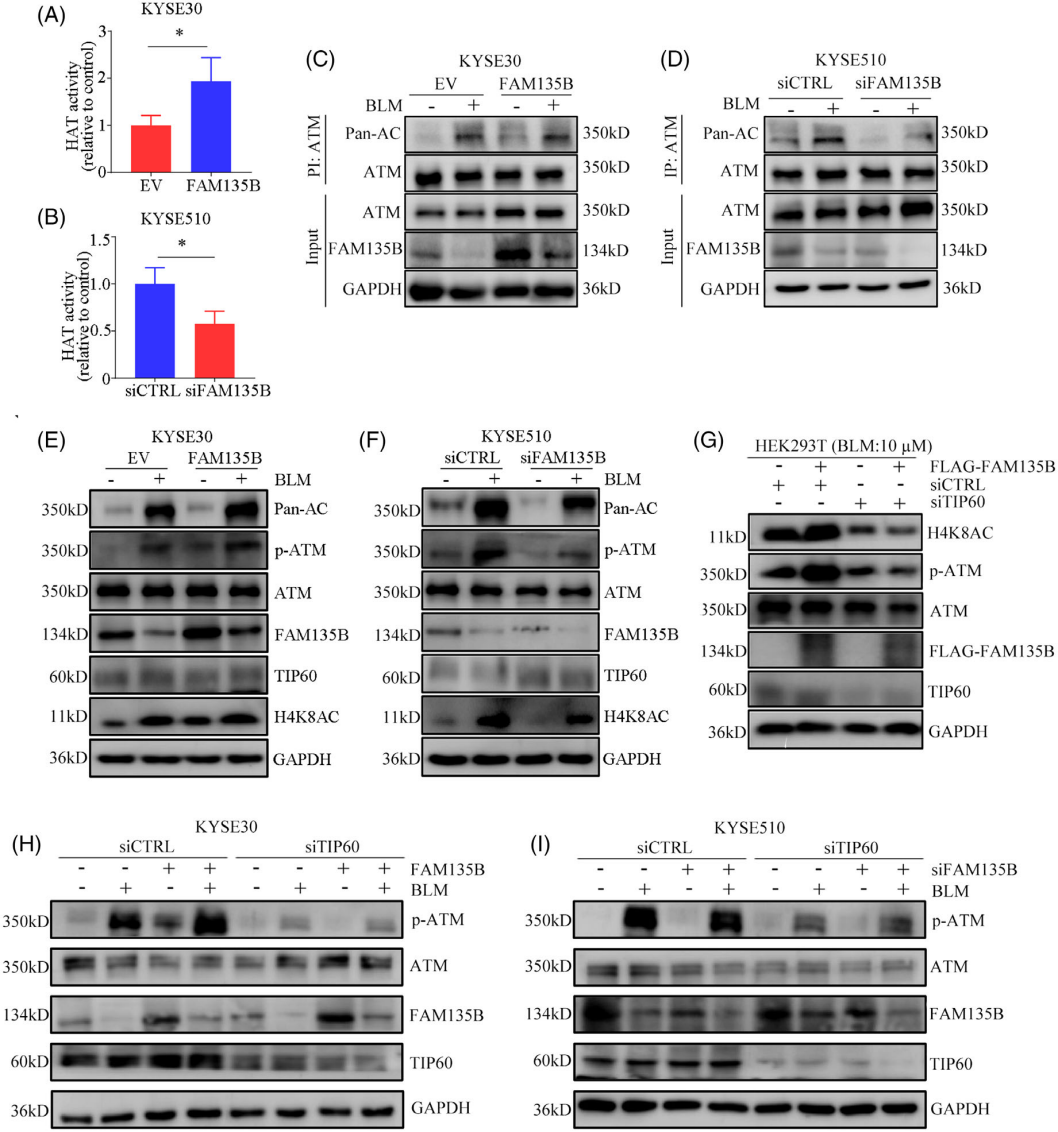
**FAM135B increases TIP60 histone acetyltransferase (HAT) activity and promotes TIP60 and ATM interactions**. (A and B) FAM135B affects the HAT activity of TIP60. KYSE30 cells (A) were transfected with FLAG‐FAM135B or empty vector and MYC‐TIP60. KYSE510 cells (B) were transfected with FAM135B (siFAM135B) or control siRNA (siCTRL) and MYC‐TIP60. Nuclear proteins were extracted from the cells treated earlier, then the protein MYC‐Tip60 complex was purified using anti‐MYC magnetic beads, and the HAT activity of Tip60 was determined by incubating 50 g of MYC‐Tip60 complex using HAT assays. The data are presented as mean ± SD. (C) KYSE30 cells overexpressed FAM135B and related controls for 48 h and were then treated with or without 10‐μM BLM for 12 h. Cell lysates were subjected to immunoprecipitation (IP) analysis. (D) KYSE510 cells were transfected with siFAM135B and related controls for 48 h, then treated with or without 10‐μM BLM for 12 h. Cell lysates were subjected to IP analysis. Pan‐AC at an identical molecular weight as ATM indicated ATM acetylation. (E and F) KYSE30 cells with overexpressed FAM135B (E) and KYSE510 cells transfected with siFAM135B (F), and their related controls for 48 h were then treated with or without 10‐μM BLM for 12 h, then cells were harvested for western blotting. Pan‐AC at an identical molecular weight as ATM indicated ATM acetylation, and H4K9AC indicated TIP60 activity. (G) HEK‐293T cells transfected with FAM135B‐FLAG, TIP60 siRNA and their controls as indicated for 48 h were treated with 10‐μM BLM for 12 h, then cells were harvested for western blot analysis. (H) FAM135B's effects on the DNA damage response (DDR) are TIP60‐dependent, KYSE30 cells transfected with TIP60 siRNA, FLAG‐FAM135B and their controls as indicated for 48 h, then treated with 10‐μM BLM for 12 h, then cells were harvested for western blot analysis. (I) FAM135B's effects on the DDR are TIP60‐dependent, KYSE510 cells transfected with TIP60 siRNA, FAM135B siRNA and their controls for 48 h were treated with 10‐μM BLM for 12 h, then cells were harvested for western blot analysis. The GAPDH antibody was used to monitor equal loading in western blots. Data are represented as the mean ± SD from three independent experiments, *p < .05.

As protein–protein interactions may affect TIP60 HAT activity, here we purified MYC‐Tip60 using anti‐MYC magnetic beads and HAT assay kits to evaluate HAT activity.^18^ Interestingly, the overexpression of FAM135B promoted TIP60 HAT activity (Figure 5A), whereas the depletion of FAM135B suppressed HAT activity (Figure 5B). These results suggested the functional effects of FAM135B on TIP60. Considering that TIP60 is an important regulator of ATM, we wondered whether FAM135B could affect the activity of ATM via TIP60. Thus, the cells with FAM135B overexpression or the control were treated with 10‐μM BLM for 12 h, and a Co‐IP assay with the anti‐ATM antibody was performed to determine ATM acetylation levels using a pan‐acetyl‐lysine antibody. Remarkably, BLM was likely to increase acetylation levels of ATM, and this effect was amplified by FAM135B overexpression (Figure 5C). In contrast, the acetylation levels of ATM were inhibited by FAM135B knock‐down (Figure 5D). For further validation, we detected ATM and pan‐acetylation levels at the same molecular weight as ATM in KYSE30 and KYSE510 cells. These results also supported the idea that FAM135B promoted ATM activation (Figure 5E,F). Additionally, the acetylation levels of H4K8AC, which indicate that the activity of TIP60 was also upregulated after BLM treatment, were positively correlated with FAM135B expression (Figure 5E,F).^42^ Furthermore, in the context of BLM‐induced genotoxic stress, we found that the overexpression of FAM135B significantly increased H4K8AC and the phosphorylation of ATM Ser1981 levels (Figure 5G). However, the overexpression of FAM135B did not apparently upregulate pATM levels following TIP60 knock‐down (Figure 5G). Moreover, we performed an epistasis analysis in ESCC cells and found no differences in p‐ATM in TIP60‐knock‐down cells regardless of FAM135B expression level (Figure 5H,I). Therefore, these results suggest that FAM135B could promote ATM activation by sustaining the TIP60 activity.

### 3.4 FAM135B promotes TIP60‐ATM pre‐assembling and is released from TIP60 for degradation upon genotoxic stress

**FIGURE 6:**
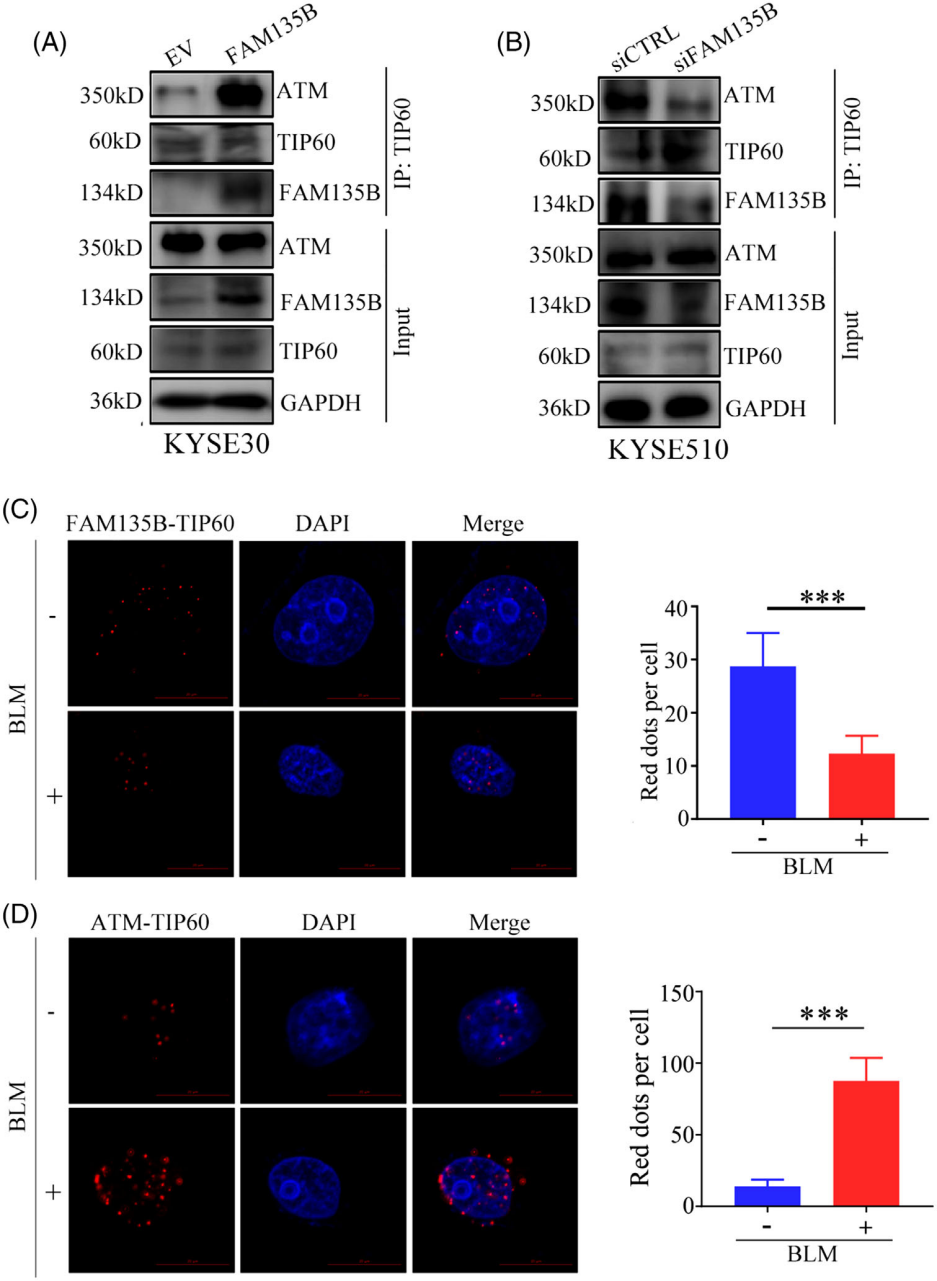
**DNA damage can decrease FAM135B and TIP60 interaction**. (A) The interaction between TIP60 and ATM after overexpression of FAM135B. KYSE30 cells overexpressed FAM135B or control vectors were subjected to co‐immunoprecipitation (Co‐IP) by anti‐TIP60 antibody and western blot was used to detect indicated proteins. (B) The interaction between TIP60 and ATM after the knock‐down of FAM135B. KYSE510 cells transfected with siFAM135B or control siRNA were subjected to Co‐IP by anti‐TIP60 antibody and western blot analysis was used to detect indicated proteins. (C) The fluorescence shows proximity ligation assay (PLA) pictures of the normal (FAM135B and TIP60) and DNA damage conditions (FAM135B and TIP60). (D) The fluorescence shows PLA pictures of the normal (TIP60 and ATM) and DNA damage conditions (TIP60 and ATM). The histograms on the right show the red spot per cell. ***p < .001

Given that FAM135B enhanced TIP60 HAT activity and promoted ATM activation, we examined whether FAM135B may contribute to TIP60‐ATM complex assembling. Based on the results of the Co‐IP assay, we found that the overexpression of FAM135B promoted the TIP60‐ATM interaction (Figure 6A), and consistently, FAM135B knock‐down attenuated this interaction (Figure 6B). We noted that during BLM‐induced genotoxic stress, FAM135B was downregulated (Figure 5G); however, after BLM treatment release, namely in the DNA damage repair period, FAM135B was upregulated (Figure 3). Considering that FAM135B binds to the chromatin binding domain of TIP60, we speculated that during DNA damage stress, FAM135B may be released from the TIP60‐ATM complex and degraded, thus enabling TIP60‐ATM to recognise the DNA damage sites immediately. To prove this, we performed a Co‐IP assay and found that the interaction between FAM135B and TIP60 weakened with the BLM treatment (Figure S3). Moreover, we used PLA experiments to verify the adjacent proximity relationship between FAM135B and TIP60 in KYSE30‐FAM135B cells. The PLA analysis identified the interaction between FAM135B and TIP60 at resting conditions. However, this interaction was significantly reduced after BLM treatment (Figure 6C). These results suggested that FAM135B maintained a specific concentration of the TIP60‐ATM preassembly module. Interestingly, the interaction between TIP60 and ATM existed at resting conditions and remarkably increased after BLM treatment (Figure 6D). However, we did not identify any apparent location changes for FAM135B via microscopy (Figure S4). These results indicated that FAM135B releases from TIP60 following DNA damage.

**FIGURE 7:**
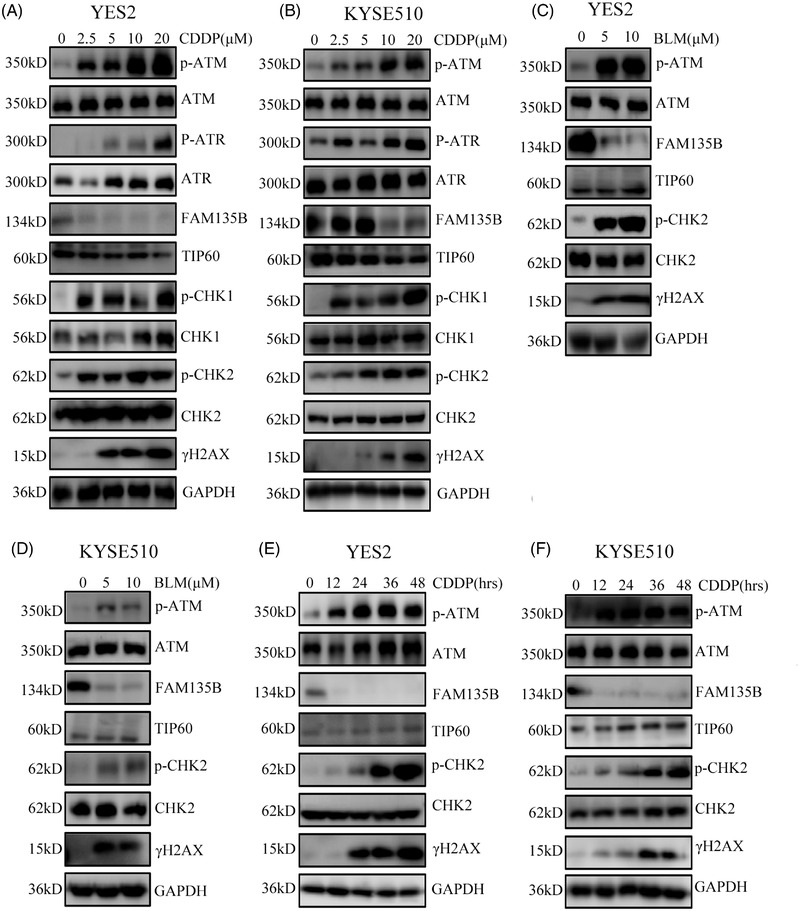
**FAM135B dramatically downregulated upon genotoxic stress**. (A–D), YES2 and KYSE510 were treated with different concentrations of CDDP (A and B) for 24 h and BLM (C and D) for 12 h and then subjected to western blot for detecting the indicated proteins. (E and F) YES2 (E) and KYSE510 (F) cells were treated with 10‐μM CDDP at various time points (0, 12, 24, 36, 48 h) and then subjected to western blot analysis for detecting the indicated proteins.

Subsequently, we determined what changes in FAM135B occurred under genotoxic insults. The data showed that FAM135B levels significantly reduced following CDDP and BLM treatments, but not in a dose‐ or time‐dependent manner (Figure 7A–F). Next, we evaluated the mRNA levels of FAM135B during the BLM treatment and found no significant changes (Figure S5). However, the DDR pathways, including ATM, ATR and CHK2, were gradually activated (Figure 7A–F), implying that the release of FAM135B from TIP60 and its subsequent degradation was critical for cells to deal with DNA damage. Altogether, it was reasonable to speculate that FAM135B might promote TIP60 interacting with ATM to form a pre‐assembly complex under normal conditions, thus sustaining a rapid and efficient DDR when the cell genome was insulted.

### 3.5 FAM135B facilitates DNA repair in vivo

**FIGURE 8:**
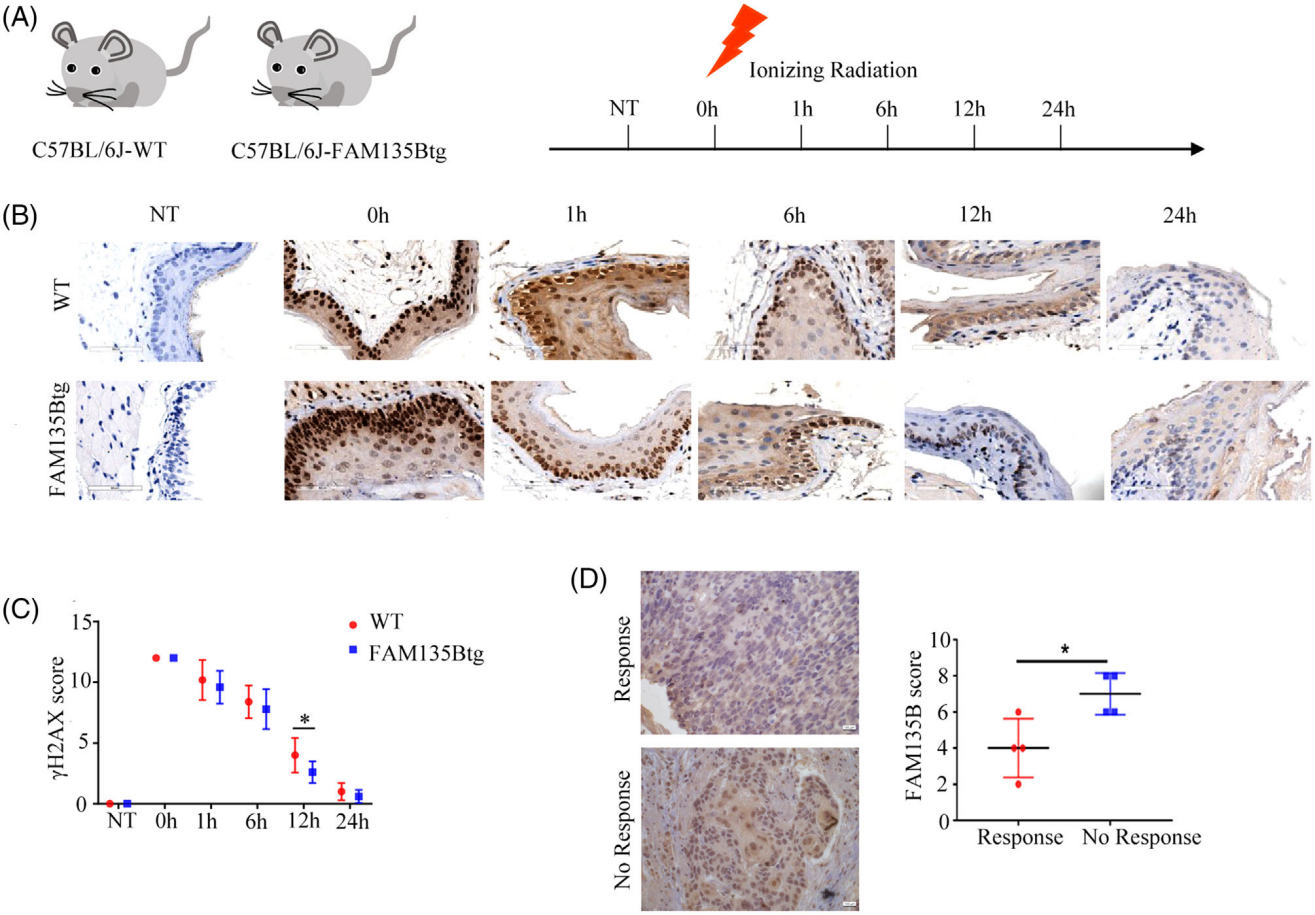
**FAM135B facilitates DNA repair in vivo**. (A) Schematic picture of mice IR assay. FAM135B transgenic (FAM135Btg) and wild‐type (WT) mice were used in this assay. In each group, one mouse was used as a negative control, five underwent 6‐Gy IR, and subsequently, mice were euthanised at the indicated time points and oesophagus tissues were taken for further immunohistochemistry (IHC) assay. (B) Representative IHC images of γpres for both FAM135Btg and WT mice, respectively. (C) Quantification of the γQuan IHC score. Five visual fields were analysed per sample. (D) Representative IHC images of FAM135B in patients with platinum‐based chemotherapy (left panel). Quantification of the FAM135B IHC score is shown in the right panel. *p < .05

To validate the in vitro observations in vivo, we used our previously constructed FAM135B transgenic (FAM135Btg) mouse model for further study. Figure 8A shows the schematic chart of the FAM135Btg mice assay. We used 6‐Gy IR to induce acute DNA damage in mice, which were then sacrificed to collect the oesophagus at different time points for the IHC assay (Figure 8B). We found no positive γH2AX staining in the non‐IR‐treated control groups. However, 6‐Gy IR caused severe DNA damage represented by strong staining of γH2AX, and γH2AX levels decreased overtime (Figure 8B,C), indicating that the DNA damage was repairable. Interestingly, the γH2AX removal rate was faster in the FAM135Btg group than in the wild‐type controls. Additionally, we collected surgery tumour tissues from patients after platinum‐based neo‐adjuvant therapy. By IHC staining of FAM135B, we found that the expression of FAM135B in the samples from resistant patients was significantly higher than that in the sensitive group, who got PR efficiency (Figure 8D), indicating that the expression levels may be implicated with platinum resistance. Taken together, these in vivo data further support the conclusion that FAM135B promotes DDR.

## 4 DISCUSSION

**FIGURE 9:**
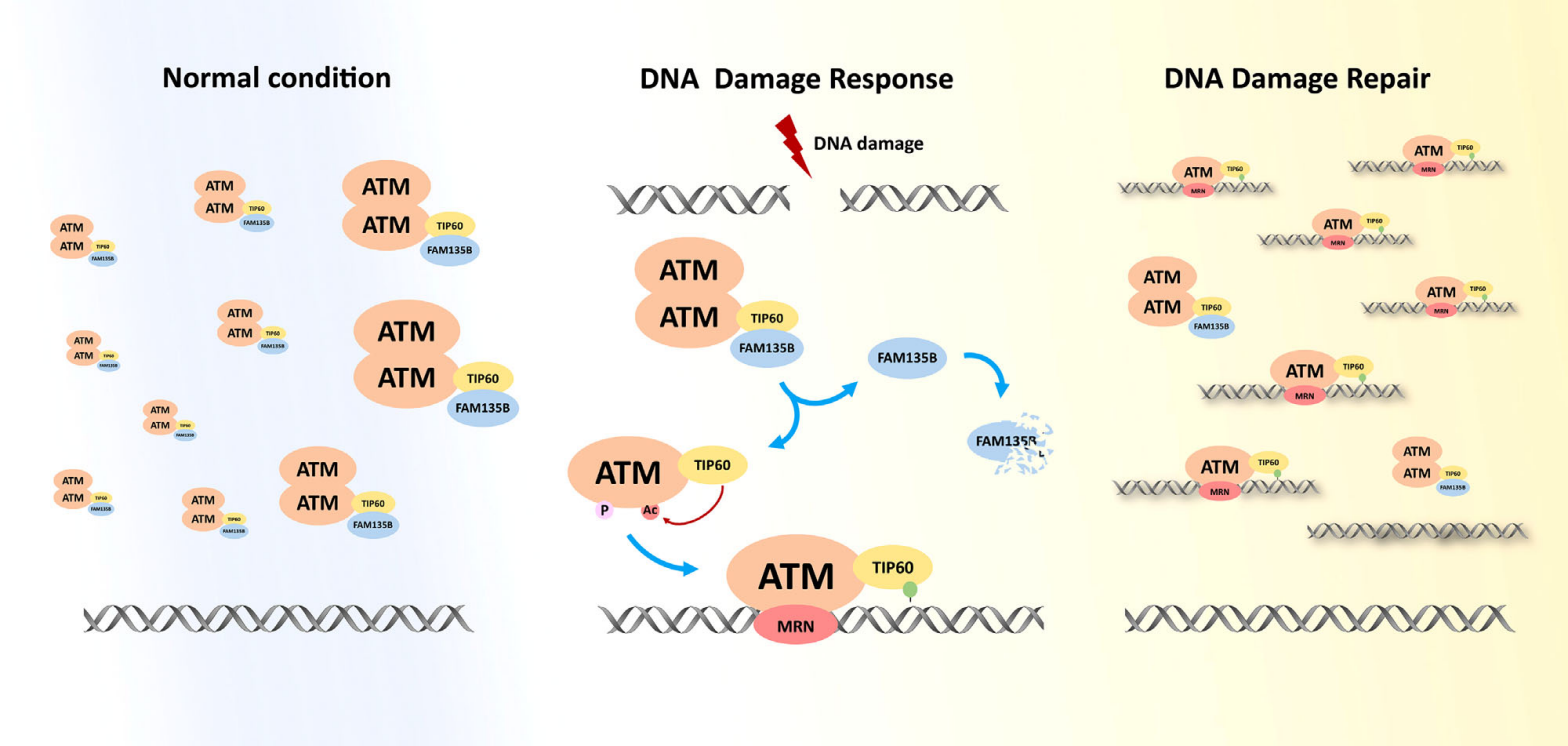
**Schematic diagram showing the model where FAM135B promotes the pre‐assemble of TIP60‐ATM under normal conditions and is released from TIP60 and degraded, thus enabling pre‐existing TIP60‐ATM assembly to be recruited to the DNA damage sites rapidly upon DNA damage**.

TIP60 acts at multiple processes in DDR and growth control. The way TIP60 rapidly responds to DNA damage and efficiently activates the downstream pathway is still largely unknown. In this study, we provided a potential mechanism by which FAM135B, a novel TIP60 regulator, enhanced the interactions between TIP60 and ATM to sustain a reservoir of TIP60‐ATM assembly under resting conditions. Once cells encounter DNA damage, FAM135B is degraded, and it releases the pre‐existing TIP60‐ATM assembly. Subsequently, the TIP60/ATM complex activates downstream pathways in response to DDR (Figure 9). We also conducted a series of comprehensive experiments to define the roles of FAM135B in promoting DDR and repair in vitro and in vivo. These revealed that the overexpression of FAM135B leads to chemotherapy resistance in vivo.

Previously, we found that the mutation and amplification of FAM135B was significantly associated with the poor prognosis of ESCC. FAM135B knock‐down is shown to reduce the growth, colony formation, migration and invasion ability of tumours.^29^ Additionally, Bi et al. showed that FAM135B suppression was sufficient to sensitise cells to IR, indicating FAM135B might play a significant role in DDR.^43^ However, the correlation between DNA damage and FAM135B is not reported, and the exact role between FAM135Bs in DDR has not been clarified. This study depicted a novel function of FAM135B in the DNA damage repair pathway. The overexpression of FAM135B protected cells from DNA damage agent BLM and CDDP in vitro and in vivo (Figure 1A,C–G), whereas the elimination of FAM135B attenuated this effect (Figure 1B–D). The IF assay for γH2AX/53BP1 and comet assay showed that FAM135B played a role in compromising DNA damage and promoting DNA repair (Figures 2 and S2A–C). Importantly, our findings revealed that FAM135B could facilitate HR and NHEJ repair in response to DNA damage (Figures 2G and S2D, E). Thus, to the best of our knowledge, this is the first study showing that FAM135B acts as a novel factor involved in DNA repair and chemoresistance.

Interestingly, Co‐IP, GST‐pulldown and IF assays demonstrated that FAM135B is bound to TIP60. As a crucial regulator of DNA injury repair, TIP60 promotes DNA repair and influences cell fate through acetylated histones, p53 and ATM.^11^ Past studies have suggested that ATF2 regulates ATM activity through TIP60 expression^26^ and that RNF8 regulates the function of TIP60, thereby facilitating efficient repair of DSB by inhibiting the activity of p53.^25^ A previous study showed that straight interactions between the ChD of TIP60 and H3K9me3 could promote the acetyltransferase activity of TIP60.^44^ Another study showed that PRMT5 was critical for the acetyltransferase activity of TIP60.^10^ Additionally, Hongmei Cui and colleagues found that the downregulation of ATF3 expression could reduce TIP60 expression and inhibit the downstream signalling pathway, leading to the accumulation of DNA damage.^18^ Although many factors are involved in TIP60 regulation, the complexity of TIP60 functions is less understood. Here, we identified FAM135B as a novel TIP60 regulator, which physically interacted with TIP60 and promoted its HAT activity. These observations add a new layer to understanding the roles of TIP60 during DNA damage.

A central mechanism for TIP60 regulating DDR is the activation of ATM kinase.^12^ Following acetylation by TIP60, ATM subsequently autophosphorylates to produce pATM Ser1981, activating various DNA repair signalling pathways.^45^ Consistent with the role of FAM135B in regulating TIP60, the knock‐down of FAM135B expression led to a reduced ATM phosphorylation. As FAM135B could promote the HAT activity of TIP60, we inferred that FAM135B may promote the ATM activity. Intriguingly, FAM135B also enhanced the interaction between TIP60 and ATM, which illustrated why upregulated acetylation of ATM was noticeable in FAM135B overexpression cells. We found that the overexpression of TIP60 could considerably upregulate pATM Ser1981, and pATM was downregulated in cells when FAM135B and/or TIP60 were depleted. We reported that the ATM activity regulated by FAM135B was TIP60‐dependent because when TIP60 was eliminated, the overexpression of FAM135B did not significantly increase ATM activity (Figure 5G). Our analysis showed that there was a loss of differences in p‐ATM in TIP60‐knock‐down cells regardless of FAM135B expression levels (Figure 5H,I).

Surprisingly, we observed that FAM135B decreased dramatically under genotoxic stress but gradually increased during the DNA damage repair period (genotoxic stress released). Based on the reduction of the TIP60/FAM135B interaction (Figures S3 and S4) and the absence of changes in FAM135B mRNA levels after BLM treatment (Figure S5), it appeared that FAM135B may be regulated by proteasomal degradation upon DNA damage. But currently, we do not know which specific factors promote the separation of FAM135B from TIP60. We speculate that the process may be affected by the drug, or possibly that it is initiated after the complex itself is activated. In addition, more studies would be useful to understand the details of the FAM135B release from the TIP60 complex. In addition, more studies would be useful to understand the regulation of FAM135B by proteasomal degradation upon DNA damage. This phenomenon was seemingly inconsistent with the observations that the overexpression FAM135B protected cells from DNA damage and promoted DNA repair presented by the rapid removal of γH2AX/53BP1 foci and shorter DNA tail moments. However, the phenomenon could be explained by the following evidence: First, FAM135B was physically bound to the ChD of TIP60; second, FAM135B enhanced the interactions between TIP60 and ATM under normal conditions; third, FAM135B promoted the HAT activity of TIP60, thereby facilitating ATM activity. Based on this evidence, we proposed that under normal conditions, FAM135B promoted the interaction of TIP60 and ATM, thus sustaining a pre‐existing reservoir of TIP60‐ATM assemblies, and ATM may be acetylated by TIP60 in this pre‐existing assembly. However, this assembly could not bind to the chromosome because the ChD of TIP60 was occupied by FAM135B. When cells were exposed to DNA damage, FAM135B was released from TIP60 and degraded quickly. Then, the TIP60‐ATM assembly was rapidly recruited to the DNA damage sites induced downstream effectors to repair damaged DNA. With the gradual completion of the DNA repair, the expression levels of FAM135B increased, maintaining the reservoir.

Nevertheless, the study has some limitations. Although we used the normal cell line (HEK293T) and mouse model, most of the conclusions were drawn from the results of tumour cell lines. Therefore, whether the molecular mechanism proposed in this study is applicable to normal cells remains to be further investigated. As altering FAM135B levels affected the cell cycle distribution, this effect may also be partly attributable to cell cycle imbalance via the AKT‐mTOR pathway caused by FAM135B silencing or overexpression.

Thus, our study identifies that FAM135B is a novel DDR regulator which physically interacts with TIP60 and promotes its HAT activity. FAM135B can also promote ATM activity through the regulation of TIP60. Most importantly, our findings provide a potential DDR mechanism where FAM135B sustains a reservoir of pre‐existing TIP60‐ATM assemblies, which can respond to DNA damage quickly under conditions of genotoxic stress. By identifying the role of FAM135B in DDR, this suggests that the expression of FAM135B in tumours may be a key in predicting patient responses to chemotherapy and radiation therapy. Therefore, FAM135B may be a target of synthetic death and combined with other DDR inhibitors may produce a synergistic effect.

## CONFLICT OF INTEREST

The authors have declared that no competing interest exists.

## Supporting information



Supplemental Tables
