# Supplementary material for: FAM135B sustains the reservoir of Tip60‐ATM assembly to promote DNA damage response
Source: Clin Transl Med. 2022 Aug 17;12(8):e945. doi: 10.1002/ctm2.945 (PMC9386324; doi:10.1002/ctm2.945)
Supplement: Supplementary file 1 — Supplemental Tables [file CTM2-12-e945-s001.docx]

Supplemental Table1: Antibodies

| Antibody | Article Number | Company |
| --- | --- | --- |
| anti-γH2AX (Ser139) | #2577 | Cell Signaling Technology |
| anti-γH2AX (Ser139) | #9718 | Cell Signaling Technology |
| anti-pATR (Ser428) | #2853 | Cell Signaling Technology |
| anti-pATM (Ser1891) | #5883 | Cell Signaling Technology |
| anti-pCHK1 (Ser137) | #2348 | Cell Signaling Technology |
| anti-pCHK2 (Thr68) | #2197 | Cell Signaling Technology |
| anti-H4K8ac | #2594 | Cell Signaling Technology |
| anti-ATM | #92356 | Cell Signaling Technology |
| anti-ATM | #2873 | Cell Signaling Technology |
| anti-ATR | #13934 | Cell Signaling Technology |
| anti-CHK1 | #2360 | Cell Signaling Technology |
| anti-CHK2 | #6334 | Cell Signaling Technology |
| anti-FLAG | #8146 | Cell Signaling Technology |
| anti-MYC | #71D10 | Cell Signaling Technology |
| anti-TIP60 | #12058 | Cell Signaling Technology |
| anti-ATM | #ET-1606-20 | Huaan Technology |
| anti-TIP60 | #10398 | Abnova |
| anti-53BP1 | ab36823 | Abcam |
| anti-FLAG | ab205606 | Abcam |
| anti-FAM135B | SAB2104963 | Sigma-Aldrich |
| anti-GST | HT601 | Santa Cruz |
| HRP-conjugated AffiniPure Mouse Rabbit IgG Light Chain | #AS061 | ABclonal |
| anti-GAPDH | #5174 | Cell Signaling Technology |
| anti-β-Actin | #3700 | Cell Signaling Technology |
| HRP-conjugated AffiniPure Goat Mouse IgG Light Chain | #AS062 | ABclonal |

Supplemental Table2: Biological agents and drugs

| biological agents and drugs | Article Number | Company |
| --- | --- | --- |
| Bleomycin | 9041-93-4 | Selleck |
| Etoposide | S1225 | Selleck |
| Cisplatin | S1166 | Selleck |

Supplemental Table3: Plasmids

| Plasmids | Article Number | Company |
| --- | --- | --- |
| pCMV-MCS-3*Flag-FAM135B | N/A | TianyiHuiyuan Life Science&Technology Inc. |
| pCMV-MCS-3*Flag | N/A | TianyiHuiyuan Life Science&Technology Inc. |
| pCMV-3Tag-3A-MYC-TIP60 | N/A | Mailgene biosciences co. ltd. |
| pCMV-3Tag-3A-MYC | N/A | Mailgene biosciences co. ltd. |
| pCMV-3Tag-3A-MYC-TIP60(1-258) | N/A | Mailgene biosciences co. ltd. |
| pCMV-3Tag-3A-MYC-TIP60(69-290) | N/A | Mailgene biosciences co. ltd. |
| pCMV-3Tag-3A-MYC-TIP60(158-395) | N/A | Mailgene biosciences co. ltd. |
| pCMV-3Tag-3A-MYC-TIP60(285-513) | N/A | Mailgene biosciences co. ltd. |
| pGEX-4T-1-GST-FAM135B | N/A | Mailgene biosciences co. ltd. |
| pGEX-4T-1-GST | N/A | Mailgene biosciences co. ltd. |
| pDRGFP | #26475 | Addgene |
| pimEJ5GFP | #44026 | Addgene |
| ISceI-GR-RFP | #17654 | Addgene |

Supplemental Table4: siRNA

| siRNA |  |
| --- | --- |
| si-FAM135B#1 | 5'-GCATTCAGCCTGTGTCCAT-3' |
| si-FAM135B#2 | 5'-GCATATGTGGATACTTCTA-3' |
| si-TIP60#1 | 5'-CCTTGACCATAAGACACTGTA-3' |
| si-TIP60#2 | 5'-CCACAGGAACUCACCACAUTT-3' |

Supplemental Table5: Primers

| Primers |  |
| --- | --- |
| human FAM135B | F: 5'-CAACAAACTCCACACGTTCC-3' |
|  | R: 5'-GTCAGCTGCAGTAGAGACC-3'  Probe: (FAM)TGGGCCTCACCTGGGAACCCTGTACAACA(TAMRA) |
| human β-actin | F: 5'-CCTGGCACCCAGCACAAT-3' |
|  | R: 5'-CTCCTTAATGTCACGCACGAT-3'  Probe: (FAM)ATCAAGATCATTGCTCCTCCTGAGCGC(BHQ1) |
| mouse FAM135B | F: 5'-TCCCCAAGACCGTTATGTGC-3' |
|  | R: 5'-GCCGCTACTTGTCATCGTCA-3' |

Supplemental Table6: Clinicopathological characteristics

| Tumor type | Sex | Age  (Y) | Clinical stage | TNM stage | Neoadjuvant therapy plan | Therapeutic effect |
| --- | --- | --- | --- | --- | --- | --- |
| ESCC | Male | 78 | IIA | ypT2N0M0 | Albumin-Bound Paclitaxel plus Nedaplatin plus Carrelizumab | Response |
| ESCC | Male | 71 | IIA | ypT2N0M0 | Albumin-Bound Paclitaxel plus Nedaplatin plus Carrelizumab | Response |
| ESCC | Male | 57 | IIB | ypT3N1M0 | Albumin-Bound Paclitaxel plus Nedaplatin plus Sintilimab | Response |
| ESCC | Male | 67 | I | Tis | Albumin-Bound Paclitaxel plus Nedaplatin plus Sintilimab | Response |
| ESCC | Male | 66 | IIB | ypT3N1M0 | Albumin-Bound Paclitaxel plus Nedaplatin plus Carrelizumab | No Response |
| ESCC | Male | 70 | IVB | ypT3N3M0 | Albumin-Bound Paclitaxel plus Nedaplatin plus Sintilimab | No Response |
| ESCC | Male | 64 | IIB | ypT3N0M0 | Albumin-Bound Paclitaxel plus Nedaplatin plus Sintilimab | No Response |
| ESCC | Male | 54 | IIIB | ypT3N2M0 | Albumin-Bound Paclitaxel plus Nedaplatin plus Carrelizumab | No Response |
